# Social Competitiveness and Plasticity of Neuroendocrine Function in Old Age: Influence of Neonatal Novelty Exposure and Maternal Care Reliability

**DOI:** 10.1371/journal.pone.0002840

**Published:** 2008-07-30

**Authors:** Katherine G. Akers, Zhen Yang, Dominic P. DelVecchio, Bethany C. Reeb, Russell D. Romeo, Bruce S. McEwen, Akaysha C. Tang

**Affiliations:** 1 Department of Psychology, University of New Mexico, Albuquerque, New Mexico, United States of America; 2 Department of Neurosciences, University of New Mexico, Albuquerque, New Mexico, United States of America; 3 Department of Psychology and Neuroscience and Behavior Program, Barnard College, New York, New York, United States of America; 4 Laboratory of Neuroendocrinology, Rockefeller University, New York, New York, United States of America; University of Minnesota, United States of America

## Abstract

Early experience is known to have a profound impact on brain and behavioral function later in life. Relatively few studies, however, have examined whether the effects of early experience remain detectable in the aging animal. Here, we examined the effects of neonatal novelty exposure, an early stimulation procedure, on late senescent rats' ability to win in social competition. During the first 3 weeks of life, half of each litter received daily 3-min exposures to a novel environment while the other half stayed in the home cage. At 24 months of age, pairs of rats competed against each other for exclusive access to chocolate rewards. We found that novelty-exposed rats won more rewards than home-staying rats, indicating that early experience exerts a life-long effect on this aspect of social dominance. Furthermore, novelty-exposed but not home-staying rats exhibited habituation of corticosterone release across repeated days of social competition testing, suggesting that early experience permanently enhances plasticity of the stress response system. Finally, we report a surprising finding that across individual rat families, greater effects of neonatal novelty exposure on stress response plasticity were found among families whose dams provided more reliable, instead of a greater total quantity of, maternal care.

## Introduction

Among social animals, dominance of some individuals over others is integral to the structure and function of a society. Such dominance is typically expressed as a hierarchy in which more dominant individuals gain greater access to desired but limited resources such as food, water, or mates compared to more subordinate individuals [1]–[4]. In contrast to field studies that reveal complex social hierarchies among animals living in natural settings [5]–[8], social competition experiments using rodents in a laboratory setting [9]–[11] have enabled researchers greater control in investigating the causes of individual differences in social dominance. Pharmacological treatments can have acute effects on success in competition [12]–[16], whereas manipulation of the neonatal environment can lead to long-lasting changes in competitive success that persist for months after the initial intervention [17]–[20]. This early programming of social dominance has been observed in postpubertal rats (∼50–90 days of age) [17]–[19] and adult rats (13 months of age) [20]. It is currently unknown, however, whether the effect of early experience on social dominance persists beyond adulthood and into old age.

For cognitive functions, correlation studies in humans support a long-lasting impact of early life environment during aging. For example, children from families of higher socioeconomic status are more likely to maintain a higher level of cognitive functioning during old age [21]–[23]. Animal experiments, which allow researchers to investigate causal relations between early environment and later functional outcomes, provide more conclusive support for a persistent effect of neonatal environment during senescence. In rats, even relatively brief and seemingly simple early life environmental manipulations can lead to changes in cognitive and brain function during senescence. Aged rats (16–24 months of age) that experienced neonatal stimulation exhibit better learning in a spatial task [24]–[26] and greater efficiency in regulation of their stress response [24; 25; 27]–[29] compared to non-stimulated controls. These findings suggest that modifying aspects of the stress response system via early environmental manipulation may lead to improved cognitive functioning during aging.

Multiple lines of evidence indicate that, among rodents, a relationship exists between the stress response system and social function, raising the likelihood that early life environment may also affect social function via its impact on the stress response system. For instance, psychological stressors [30]–[32] or administration of stress hormones [33]; [34] affect aspects of social dominance such as aggressiveness and success in competition for limited resources. Furthermore, dominance status has been found to correlate with levels of stress hormone release [35]–[38]. These findings suggest that early life stimulation, which is known to produce long-lasting changes in the stress response system, may result in permanent changes in social function that are observable even during senescence. In the present study, we examine whether neonatal novelty exposure [39]; [29]; [20], an early life stimulation procedure, affects success in social competition as well as post-competition circulating stress hormone concentration among late senescent rats. As individual differences in maternal care have been shown to be associated with offspring's hypothalamic-pituitary-adrenal (HPA) axis development [40], we also explore whether differences in maternal care influence the effect of neonatal novelty exposure on competitive success and stress response function.

## Results

**Figure 1:**
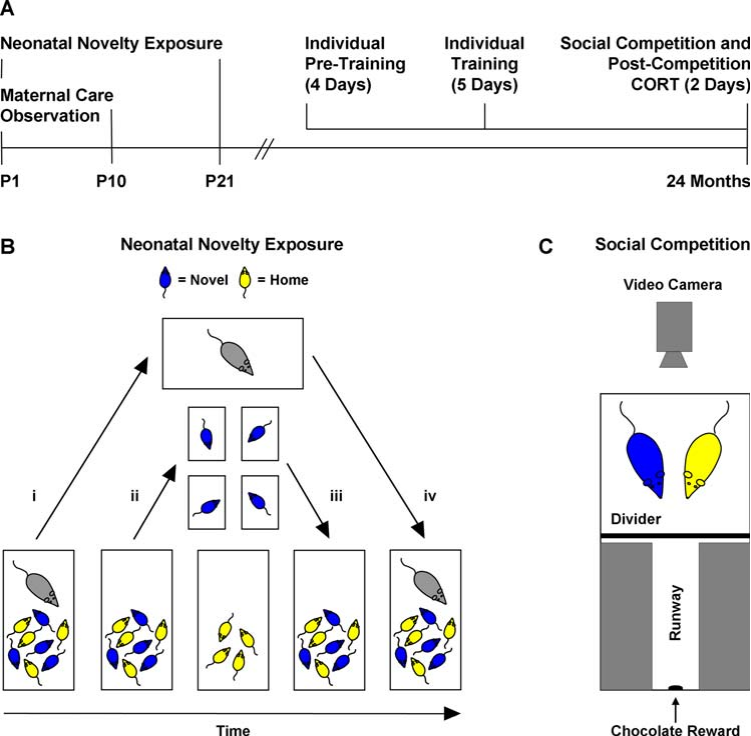
Experimental methods. A. Timeline. B. Sequential steps in carrying out the within-litter neonatal novelty exposure procedure: (i) Dam is removed from the home cage; (ii) Novel pups are transferred to individual non-home cages and yoked Home pups receive a matching amount of experimenter contact; (iii) After 3 min in the non-home cages, Novel pups are returned to the home cage in which the Home pups remain; (iv) Dam is returned to the home cage. C. Apparatus used to assess rats' ability to compete against a conspecific for exclusive access to chocolate rewards. Note that the runway was sufficiently narrow as to allow only one rat at a time to fully enter.

Neonatal novelty exposure was performed from postnatal day 1 to 21. Briefly, half of the pups from each litter were exposed to a novel cage for 3 min a day (Novel) while the other half remained in the home cage (Home; Fig. 1B). During this procedure, amount of experimenter contact and duration of separation from the dam were matched between Novel and Home pups, isolating exposure to a novel environment as the critical difference between the two groups. After weaning on postnatal day 21, Novel and Home rats were permanently housed in individual cages.

**Figure 2:**
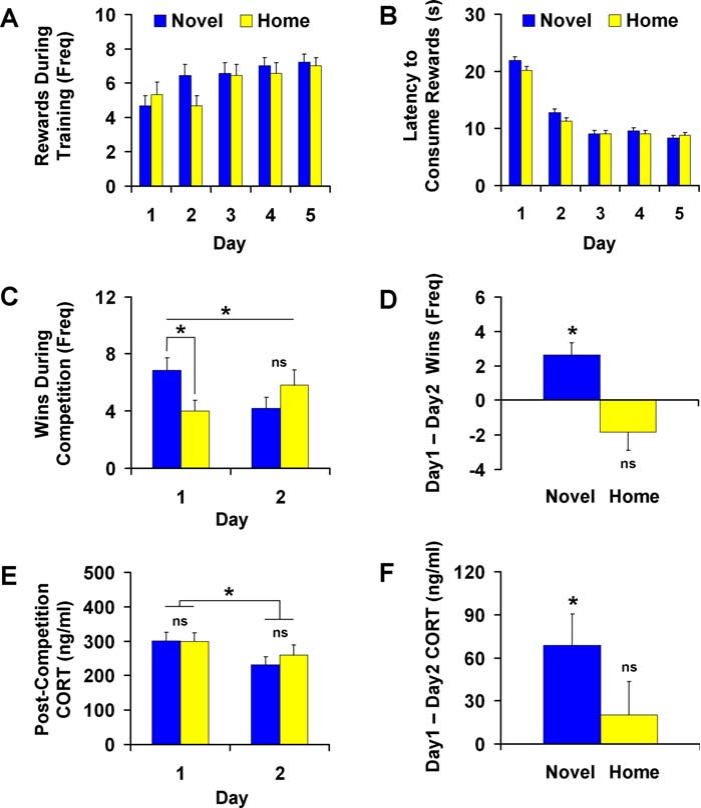
Permanent effects of neonatal novelty exposure on social competitive success and stress response system function (24 months of age). AB. When trained individually, Novel and Home rats showed no difference in learning to obtain chocolate rewards nor did they differ in final level of performance (*N*_Novel_ = 11, *N*_Home_ = 11). C. During paired social competition testing, Novel rats won significantly more rewards than Home rats on Day 1 but not on Day 2 (*N* = 11 pairs of Novel and Home rats). D. Novel but not Home rats exhibited a significant reduction in wins from Day 1 to Day 2. E. Despite a significant difference between Novel and Home rats in wins on Day 1, there was no parallel difference in post-competition corticosterone (CORT) concentration. Overall, CORT response to social competition significantly decreased across testing days (*N* = 14 pairs of Novel and Home rats). F. Novel but not Home rats exhibited significant habituation of CORT response across days. In all panels, data are mean±SEM; * indicates *p*<0.05; ns indicates *p*>0.05.

When Novel and Home rats reached 24 months of age (Fig. 1A), their ability to compete against a conspecific for limited access to chocolate rewards was assessed in a social competition task. Prior to social competition, rats were individually trained to enter a narrow runway leading to chocolate rewards (Fig. 1C). Across five consecutive days of training, the number of rewards consumed increased (*F*(4,24) = 13.23, *p*<0.001; Fig. 2A) and the latency to begin chocolate consumption decreased (*F*(4,24) = 20.86, *p*<0.001; Fig. 2B). On the last day of individual training, there were no significant differences in performance between Novel and Home rats in terms of both number of rewards consumed (*p* = 0.710) and latency to consume the rewards (*p* = 0.876). The fact that Novel and Home rats exhibited similar levels of performance throughout training indicates that the groups did not differ in either motivation or proficiency in obtaining the chocolate rewards prior to dyadic competition. Furthermore, Novel and Home rats did not differ in their levels of general activity as measured by their spontaneous activity prior to daily training (*p*s>0.505).

On the 2 consecutive days immediately after training, pairs of Novel and Home rats competed against one another for exclusive access to chocolate rewards (Fig. 1C). Competition testing occurred in neutral, non-home cages. Rats within pairs were matched such that within-pair differences in final training performances and body weights were not statistically significant (training performance: *p* = 0.167; weight 2 months prior to competition: *p* = 0.670; weight 1 month after competition: *p* = 0.441). Novel and Home rats differed in their winning patterns across the two days of competition testing (Novelty by Day interaction: *F*(1,9) = 6.85, *p* = 0.028; Fig. 2C). On the first day of competition, when the testing situation was novel due to the unexpected presence of a competitor, Novel rats won significantly more rewards than Home rats (*t*(10) = 1.82, *p = *0.0495; Fig. 2C). This competitive advantage of Novel over Home rats was unlikely caused by a difference in speed of reaching the rewards, as latencies did not differ between groups (Novel: 5.95±3.12 s; Home: 6.60±3.46 s; *p* = 0.611). On the second day of competition, when the testing situation was no longer novel, Novel and Home rats did not differ in number of rewards won (*p* = 0.336, Fig. 2C). This change in competitive success can be presented as a difference score (Day 1 wins–Day 2 wins). Using this score, we found that the number of wins by Novel rats decreased across days (*t*(10) = 3.68, *p = *0.004; Fig. 2D) whereas the number of wins by Home rats showed no significant change across days (*p* = 0.120; Fig. 2D).

To investigate possible neuroendocrine mechanisms contributing to this difference in competitive success, we measured circulating corticosterone (CORT) concentration 15 min after competition (∼30 min after the onset of competition testing). On both days, competition testing resulted in a clear increase in CORT levels in comparison to basal levels we have previously observed among aged rats (110.50±9.59 ng/ml) [29]. Notably, on the first day of competition—when Novel and Home rats showed a significant difference in competitive success—no group difference was found in post-competition CORT (compare Fig. 2C and 2E). Across the two testing days, CORT levels showed a significant overall reduction (*F*(1,12) = 7.13; *p = *0.020; Fig. 2E), Importantly, this CORT habituation was significant only for Novel rats (*t*(14) = 3.18, *p* = 0.007; Fig. 2F) and not for Home rats (*p* = 0.411; Fig. 2F). This contrasting pattern between Novel and Home rats in CORT habituation across days mirrors the pattern of competitive success across days, with Novel rats alone showing a decrease in both CORT release and competitive success in response to a reduction in the novelty of the testing situation (compare Fig. 2D and 2F).

**Figure 3:**
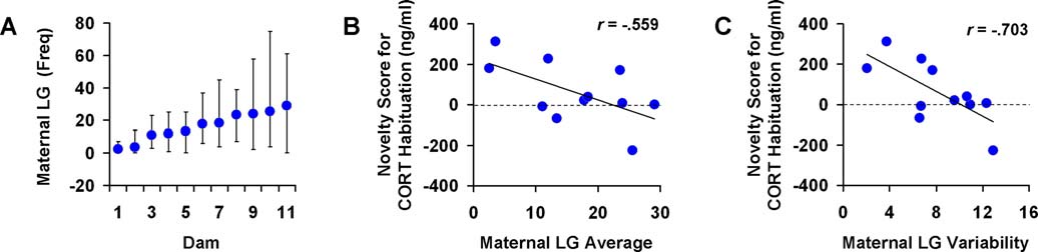
Maternal care during brief 10-min windows immediately after repeated novelty exposure predicts the effect of novelty exposure on CORT habituation among aged offspring. A. Greater average amount of maternal licking and grooming (LG) was associated with greater day-to-day variability in maternal LG (dots and bars indicate average and range, respectively, of LG across days for individual dams; *N* = 11 litters). B. Greater average amount of maternal LG was associated with negative novelty scores for CORT habituation (marginally significant). D. Smaller day-to-day variability in maternal LG was significantly correlated with positive novelty scores for CORT habituation.

To investigate the contribution of individual differences in post-novelty exposure maternal care to the observed social and neuroendocrine differences between Novel and Home rats, we measured both discriminative and non-discriminative maternal care during the first 10 postnatal days. Discriminative maternal care was measured by dams' priority of retrieval of Novel and Home pups immediately following the neonatal novelty exposure procedure. Non-discriminative maternal care was measured by dams' licking and grooming (LG) [41]–[43] of all her pups regardless of Novel versus Home identity after they were retrieved and returned to the nest. Similar to previous findings [20], we found no differences between Novel and Home pups in either retrieval latency (*p* = 0.685) or retrieval order (*p* = 0.928). Therefore, we have no evidence that maternal discriminative treatment mediated the effects of neonatal novelty exposure on competitive success or CORT habituation. Analysis of maternal LG irrespective of Novel or Home identity revealed large individual differences across dams in both the average amount of LG (dots in Fig. 3A) and variability of LG (vertical bars in Fig. 3A) across postnatal days. The dams with higher average LG also showed higher day-to-day variability in LG (Fig. 3A), raising the possibility that high levels of post-stimulation maternal care may not always be associated with enhanced offspring function and that *reliability* may be more important than *quantity* of maternal care.

This led us to test two related but distinct hypotheses concerning the nature of maternal influence on social competitive ability and HPA plasticity: (1) greater *average amount* of post-novelty exposure maternal care is associated with larger novelty exposure effects on behavior and HPA function, and (2) greater *reliability* (less variability) of post-novelty exposure maternal care is associated with larger novelty exposure effects. Effects of neonatal novelty exposure on individual litters were measured by novelty scores, defined as differences between the Novel mean and the Home mean for each litter. Two separate novelty scores were used—one for competition wins on the first day of testing and one for CORT habituation across the two testing days. A positive or negative novelty score means that the effect of novelty exposure was an increase or a decrease in the dependent measure, respectively.

We first considered the average amount of post-novelty exposure maternal LG as a predictor of novelty exposure effects. We found no evidence that average maternal LG was correlated with novelty scores for competition wins (*r* = −0.184, *p* = 0.694, *n* = 7), and we found a marginally significant but negative correlation between the average amount of maternal LG and the novelty scores for CORT habituation (*r* = −0.559, *p* = 0.051, *n* = 11; Fig. 3B). This lack of positive correlation indicates that greater average amounts of post-stimulation maternal care were not associated with greater enhancements in social and neuroendocrine development among novelty-exposed pups.

As a greater amount of maternal care may be indicative of higher variability—hence lower reliability—of maternal care, we considered the day-to-day variability of maternal care as an alternative predictor of novelty exposure effects. We found a significant negative correlation between LG variability and the novelty scores for CORT habituation (*r* = −0.704, *p* = 0.016, *n* = 11; Fig. 3C) and a negative but non-significant correlation for competition wins (*r* = −0.302, *p* = 0.511, n = 7; see comment in Data Analysis section). These results suggest that when post-novelty exposure maternal care is delivered unreliably, more care may result in a reduction in the effect of neonatal novelty exposure on offspring HPA plasticity, whereas when maternal care is delivered reliably, less care can result in more positive effects of neonatal novelty exposure.

We further tested for correlations between the male-female composition of litters and maternal care and offspring measures. We found no evidence that any of the dependent measures were significantly related to litter composition (*p*s>0.232).

## Discussion

Following rats from birth to late senescence, we examined the effect of neonatal novelty exposure, an early life stimulation procedure involving daily 3-min exposures to a novel environment for the first 3 weeks of life, on success in competition against a conspecific for exclusive access to chocolate rewards. In the absence of a competitor, novelty-exposed and home-staying rats displayed similar levels of spontaneous activity and achieved similar levels of performance in terms of number of rewards obtained and latency to obtain the rewards. In the presence of a competitor, however, novelty-exposed rats won more rewards than home-staying rats on their first but not the second day of testing. No difference in post-competition circulating CORT concentration was found between novelty-exposed and home-staying siblings on either day of testing. Instead, among novelty-exposed but not home-staying rats, a reduction in CORT concentration was observed across the two testing days. This between-sibling novelty effect on CORT habituation among aged rats was negatively correlated with the variability of maternal care received immediately after daily novelty exposures during infancy.

### Permanency of the neonatal novelty exposure effect on social competition

Over the past half of a century, numerous studies have investigated the effects of neonatal experience on psychological and physiological function in later life [44]–[47]. Although most of these studies have examined relatively short-term effects of neonatal experience (i.e. among adolescent and early adult animals), few have examined the effects of neonatal experience across the entire lifespan (i.e. among senescent animals) [24]; [25]; [27]; [28]; [48]; [26]; [29]; [49]. Remarkably, three studies that followed rats from birth until 18 months [26] and 24 months of age [24]; [25] revealed that early stimulation has a permanent effect on spatial learning, even though such experience involved no more than ∼15 min of daily separation from the dam and exposure to a non-home environment along with necessary experimenter handling.

One key characteristic of this early experience effect is that it was observed in the Morris water task—a task involving negative reinforcement in which behavioral responses are required to terminate exposure to cold water. Early literature on neonatal stimulation, however, noted that the effects of early experience on performance in tasks involving negative reinforcement do not necessarily generalize to tasks involving positive reinforcement, such as those in which responses are required to obtain food [50]; [see 39]. Therefore, the effect of early experience on learning in the Morris water task does not necessarily generalize to an effect on ability to obtain rewards in the presence of a competitor. Only a direct investigation of competitive success during senescence can allow the determination of whether early experience via simple stimulation protocols can impact this social function throughout the entire lifespan.

The present study directly investigated the effects of neonatal stimulation on competitive success among senescent rats. By training both novelty-exposed and home-staying rats until they reached asymptotic performance, we were able to separate the effect on competitive success from an effect on learning to locate the chocolate reward in the testing environment. By matching within-pair training performances and body weights, we were able to rule out motivational and body size differences as potential confounding factors. With these control measures taken, we found that senescent rats that experienced 3-min daily exposures to a non-home environment during infancy exhibited a greater number of wins in competition against a conspecific for access to a desired resource compared to control rats that stayed in the home environment. This finding provides a direct demonstration that early stimulation can lead to enhanced success in social competition among senescent rats, suggesting that the effect of early experience among aged rats can be generalized from tasks involving negative reinforcement to those involving positive reinforcement. This finding also extends previous findings of an effect of early stimulation on social competition from postpuberty [17]–[19] and adulthood [20] to late senescence.

### Context-dependent expression of the neonatal novelty exposure effect

Behavioral expressions of social dominance are known to be context-dependent. For instance, when two or more unfamiliar rats are introduced to each other, there is typically an initial period of fighting that disappears within a few minutes or hours [51]; [52]. It is speculated that such initial aggressive behavior serves to establish a dominance hierarchy that, once established, renders further aggressive encounters between individuals unnecessary [51]. In the present study, we found that success in competition against a conspecific for resources may also depend on the context of the social encounter. That is, novelty-exposed rats were found to win more often than home-staying rats only during the first day of competition testing, with the two groups showing comparable numbers of wins on the second day of testing. This observation suggests that the neonatal novelty exposure-induced increase in competitive success may be dependent upon the novelty of the social situation and that a modification of novelty response may underlie the observed difference between the Novel and Home rats in competitive success.

Context-dependent effects of neonatal novelty exposure across other functional domains have been previously observed in studies from independent cohorts of rats. In the open field, a novelty exposure effect on measures of emotional reactivity was most pronounced during the initial trials [53]. In the Morris water task, a novelty exposure effect on CORT release was found for an unexpected stressor (a surprising exposure to an open field between swim trials) but not for an expected stressor (normal daily swimming routine) [20]. In a test for functional brain asymmetry, a novelty exposure effect on spontaneous turning preference was observed only during the first day of exposure to a novel testing environment but not during the second day [54]. Together, these converging findings suggest that the diverse expressions of the effect of neonatal novelty exposure across different functional domains share at least one common underlying mechanism—a differential regulation of physiological and emotional response to novelty.

### Effect of neonatal novelty exposure on HPA plasticity

The observation that novelty-exposed and home-staying rats differed in competitive success during senescence implies that neonatal novelty exposure must have induced permanent changes within the brain. It is interesting to speculate what these changes might be. Previous studies report that senescent rats that experienced neonatal stimulation differ from non-stimulated rats in HPA negative feedback efficiency [24]; [25]; [27]; [28]; [26]; [29] and neuromodulation within the amygdala [49] and neocortex [48]. As many of these effects of early stimulation involve the stress response system, it is possible that neonatal novelty exposure may have affected competitive success among the senescent rats via a permanent modification of HPA function. The present assessment of circulating CORT concentration shortly after the social competition testing showed that CORT levels were elevated relative to the basal levels we have previously observed among aged rats [29], confirming that the experience of social competition involves a change in the state of the HPA axis.

Surprisingly, in contrast to studies showing that individual differences in aggression are associated with differences in CORT concentration [55]–[57], the novelty exposure effect on competitive success was not accompanied by a novelty effect on circulating CORT concentration. Instead, we observed a significant reduction in CORT level across the two days of testing, i.e. habituation of CORT response to social stress, among novelty-exposed but not home-staying rats. This habituation of the HPA response to social stress expressed selectively among Novel rats is consistent with a previous finding of habituation to repeated cold stress among handled but not non-handled rats [66]. These findings suggest that differences in early life experience may contribute to individual differences in the plasticity of the HPA axis long after the initial early experience. Furthermore, our finding among aged rats demonstrates that an enhancement of HPA plasticity can persist into old age. As both humans [58]–[61] and non-human animals [62]–[65] exhibit habituation of stress hormone release to repeated stressors, early environmental characteristics that affect such habituation may be important for our understanding of individual differences in coping with social as well as non-social stress.

Functionally, a habituation of CORT response to familiar stressors can lead to a cumulative reduction in the overall amount of CORT release and, consequently, a reduction in the cumulative exposure of neural tissue to this stress hormone. Furthermore, differences in this cumulative exposure to CORT can lead to differences in hippocampal glucocorticoid receptor concentration, which is critical for regulation of HPA function [67]. In an *in vitro* electrophysiological study, novelty-exposed rats showed greater suppression of hippocampal population spikes at high CORT concentrations than home-staying rats [68], implying that more functional glucocorticoid receptors were available among novelty-exposed rats to mediate this differential suppression. Because high levels of circulating stress hormones are known to result in reduced synaptic plasticity [69] as well as brain atrophy and cognitive dysfunction [70]–[72], less stress hormone release in response to familiar stressors may ultimately promote greater brain and cognitive function. Therefore, our finding of CORT habituation to a repeated stressor among novelty-exposed rats may offer an explanation for why rats that experienced neonatal novelty exposure show enhanced synaptic plasticity [73]; [74], faster acquisition of a spatial task [39]; [20], and longer retention of memories for a social partner [29]; [74] and an odor discrimination task [39] in comparison to home-staying rats.

### Maternal modulation of offspring HPA plasticity during senescence

In contrast to other neonatal stimulation studies that assign entire litters of pups to stimulated versus control conditions (e.g. in neonatal handling studies [44]–[47]), here the stimulated (novelty-exposed) and control (home-staying) pups shared the same dam. Therefore, the differences in social and neuroendocrine function between novelty-exposed and home-staying rats cannot be mediated by maternal individual differences. As physical contact alone with an anesthetized dam after neonatal stimulation is sufficient for facilitating recovery of pups' stress response in the absence of any active maternal care [75], it is unlikely that preferential maternal care toward novelty-exposed pups could be the sole cause of the observed long-lasting enhancements. Our analysis of discriminative maternal care behavior immediately upon pup-dam reunion—a time when discriminative treatment is most likely to occur—revealed a lack of differences in retrieval latency and order between novelty-exposed and home-staying pups. As pups that are retrieved faster after nest disturbance also receive more around-the-clock active nursing from the dam [20], this lack of difference between novelty-exposed and home-staying pups in retrieval measures further questions the likelihood that preferential maternal care is the cause of the observed novelty effects on social and neuroendocrine function.

In the absence of any evidence supporting differential maternal care between stimulated and control pups (i.e. maternal mediation), we consider the possibility that the dam modulates the effect of neonatal novelty exposure. As physical contact between the dam and pups suppressed handling- and shock-induced CORT response [76], it is possible that by providing different amounts of physical contact upon reunion, dams can differentially affect the time course of pups' CORT response across different litters, thereby modulating the physiological as well as the psychological effects of the otherwise uniformly applied novelty exposure procedure. Surprisingly, the observation of a negative correlation between the average maternal LG and the novelty score for CORT habituation failed to confirm this speculation. Higher levels of post-novelty exposure maternal care appeared to be associated with smaller novelty exposure-induced enhancements in HPA plasticity. Although somewhat counterintuitive, this finding is consistent with a repeatedly observed dissociation between higher levels of maternal care behavior and early stimulation-induced enhancements in offspring HPA function found in studies of several mammalian species, including rats [77]–[79], rabbits [80], and non-human primates [81] (see brief review in [20]). To explain functional differences in the offspring, investigators of those studies attribute sources of influence to factors other than maternal care, such as a direct stimulation effect via the handling procedure [77]; [80], separation from the dam [79], or stress activation [81], or to an interaction between maternal care and environmental stress [82].

Our present finding concerning post-stimulation maternal care begs the question of why higher levels of maternal care should be associated with less of a stimulation effect. This observation would make sense if one accepts the possibility that maternal care can be a source of either comfort or stress depending on its predictability or variability. High levels of sporadically delivered maternal care may not facilitate or may possibly retard recovery of pups' HPA response to neonatal stimulation, whereas lower levels of reliably delivered care may be more effective at facilitating such recovery. This hypothesis is confirmed in the present study by a negative correlation between the day-to-day variability of maternal LG and the within-litter novelty scores for CORT habituation. The result showed that the less variable (i.e. more reliable or predictable) the maternal care after daily neonatal novelty exposure, the greater the effect of neonatal novelty exposure on offspring's HPA plasticity, thus supporting the maternal modulation hypothesis, which states that activation of pups' HPA axis and maternal behavior exert converging influence in shaping the long-term development of HPA function [20].

### Conclusions

By following rats from birth to late senescence, we found that rats that experienced 3-min daily exposures to a novel environment for the first 3 weeks of life exhibited greater ability to win in social competition than their siblings that stayed in the home cage and, remarkably, that this enhanced competitive success was detectable during old age. This enhanced competitive success among novelty-exposed rats was accompanied by increased plasticity of HPA function. Furthermore, the effect of neonatal novelty exposure on HPA plasticity was modulated by the reliability but not the average amount of post-novelty exposure maternal care. These findings support the view that differences in the neonatal environment can have profound life-long impact on social and HPA function and that this impact is modulated by differences in maternal care reliability. This view that early experience and maternal care exert converging influences on offspring development stands in contrast to an alternative view that neonatal stimulation exerts no direct effects on pups but, rather, that maternal care solely mediates the effects of neonatal stimulation on adult functional outcome.

## Materials and Methods

### Animals

All experimental procedures were approved by the Institutional Animal Care and Use Committee at the University of New Mexico and were in accordance with the NIH Guide for the Care and Use of Laboratory Animals. Twelve pregnant Long Evans dams (Harlan, Indianapolis, IN) arrived at the vivarium 10 days before giving birth. The day of birth was designated postnatal day 0 (P0). Within 24 hours of birth, litters were culled to 8 pups, keeping as many males as possible; females were kept only to maintain equivalent litter sizes. After culling, the number of males in each litter ranged from 3 to 8, and the number of females ranged from 0 to 5. Weaning occurred on P21. Thereafter, rats were individually housed in translucent plastic cages (51×25×22 cm) and maintained on a 12-hr light/dark cycle (lights on at 0800 hr) with food and water *ad libitum*. Temperature and humidity were maintained at 21°C and 25%, respectively.

A total of 30 male offspring participated in the present experiment, which spanned the rats' lifetimes (Fig. 1A). During infancy, neonatal novelty exposure was performed and observations of post-novelty exposure maternal care were made. During senescence, rats were individually trained to obtain chocolate rewards and then tested for their ability to obtain the rewards in the presence of a competitor; measures of post-competition circulating CORT concentration were also obtained. Throughout individual training, social competition testing, blood collection, and CORT assay, experimenters were blind to rats' group identities. Furthermore, the temporal orders during training and testing, blood collection, and sample processing were counterbalanced between Novel and Home groups.

### Neonatal Novelty Exposure

On P1, half of the pups from each litter were pseudorandomly assigned to the Novel group and the other half to the Home group such that each group contained pups of roughly matched body weights. Group membership was distinguished via patterns of toe tattoos (left first digit/right fifth digit or left fifth digit/right first digit), with different patterns counterbalanced between Novel and Home groups. Neonatal novelty exposure (Fig. 1B) was conducted daily in the housing room from P1 to P21. First, the dam was removed from the home cage and placed in a separate holding cage in the housing room. Next, Novel pups were placed individually in novel, non-home cages lined with fresh bedding of the same type as that used in the home cage. After 3 min in the novel cages, Novel pups were returned to the home cage in which the Home pups remained. Every time a Novel pup was picked up by the experimenter and transferred into or out of a novel cage, a yoked Home pup was similarly picked up and returned to the home cage, thus matching amount of experimenter contact between groups. Only after the Novel pups were reunited with the Home pups was the dam reunited with all her pups, thus matching amount of maternal separation between groups.

### Maternal Care Behavior

On P1-10, immediately after the return of the dam to the home cage after novelty exposure, maternal behavior in the home cage was videotaped for 10 min. At the end of novelty exposure, Novel and Home pups were placed in separate compartments of an open-top plastic container so that discriminative maternal behavior toward Novel and Home pups could be measured in terms of pup retrieval preference (for details, see [20]). Retrieval latency for each pup was defined as the time delay from the onset of the observation to the first time the pup was picked up by the dam. We also recorded the dam's first choice as a binary variable indicating whether a Novel or Home pup was retrieved first. As a measure of nondiscriminative maternal care, frequency of maternal licking and grooming (LG) was measured during the 10-min observation window in 5-s increments. This measure was considered nondiscriminative because dams tend to keep pups in a pile in the nest after retrieval, making it impossible to accurately measure LG directed toward individual pups. If LG was present any time during each increment, an occurrence of 1 was counted. To obtain an estimate of inter-rater reliability, LG on one of the 10 days was observed by two coders. A score of *r* = 0.89 was obtained. To measure day-to-day variability in LG, we removed the systematic increasing trend due to habituation of the dam to the novelty exposure procedure by fitting a straight line through each dam's daily LG and keeping the residuals for each of the 10 days. The standard deviation of these daily residuals was computed for each dam as its variability index. Nursing of pups rarely occurred during the 10 min immediately after the disturbance of the novelty exposure.

### Social Competition

#### Apparatus

To assess ability to compete against a conspecific for exclusive access to a reward, we designed and built a testing apparatus (25×25×22 cm) that required rats to enter a narrow runway—into which only one rat could fully enter—to consume a chocolate reward located at the end wall of the runway (Fig. 1C). The apparatus was comprised of two opaque walls attached to a roof. The space between the two walls formed a runway that was half the length of the testing cage. A black roof was used to keep the runway dark, thereby increasing the likelihood of rats entering the runway upon their first encounters with the apparatus. One end of the runway was open and the other blocked by a third wall made of transparent Plexiglas with all but a small window area covered with black tape. A small drop of melted chocolate was applied to the center of this window during each trial. The chocolate drop was visible to the rat inside as well as to the experimenter observing from outside. The apparatus was designed to be transferable between testing cages, as one apparatus was used for the testing of all animals in different cages.

#### Pre-training in the home cage

To familiarize rats with the chocolate rewards, a small amount of melted chocolate (Hershey's Milk Chocolate Chips) was applied with a Q-tip to the front wall (nearest to the experimenter) inside of the home cages in the home room once a day for 4 days. On the last day of pre-training, most rats consumed the chocolate immediately and all rats consumed the chocolate within 1 min. It is important to point out that this immediate response occurred even when rats had constant free access to standard rat chow (Harlan Teklad).

#### Training to obtain chocolate rewards without competition

Rats were trained individually on 5 consecutive days to enter the runway and consume a small drop of melted chocolate at the end of the runway. Training was conducted in a non-home testing room but within rats' own home cages. Both pre-training and the use of home cages in the training phase were designed to facilitate learning, thus minimizing training duration. On each day, rats were first habituated to the training environment for 2 min while being confined to one-half of the cage by an opaque divider. At the beginning of each of the subsequent 8 trials, a drop of chocolate was applied to the window on the rear wall of the runway, and the apparatus was placed into the cage behind the divider (see Fig. 1A). Next, a brief tone was sounded to signal the removal of the divider, which allowed the rat access to the chocolate. The trial was terminated either when the rat consumed the reward or when the 30 s upper limit was reached. Between trials, the apparatus was wiped clean with a paper towel to remove any residual chocolate before applying a new drop. On the first day of training, a maximum trial duration of 60 s was used for the first trial. Rats were trained until they reached asymptotic performance (i.e. until the daily number of rewards obtained plateaued for 3 consecutive days). The latency to begin consuming the reward was recorded for each trial. If a rat did not consume the chocolate, a latency equal to the maximum trial length was recorded.

#### Measurement of general activity

Activity levels were measured during the 2-min habituation sessions that preceded each day of individual training. During the habituation sessions, rats were confined to one-half of the testing cage, limiting their spontaneous movements to rears and discrete right and left turns. Activity level was measured by summing the frequencies of rears, right turns, and left turns. A rear was defined as the rat rising up on its hind legs. A right or left turn was defined as a cumulative 90° rotation of the rat in a clockwise or counterclockwise direction, respectively.

#### Social competition testing

Dyadic competition was set up between Novel and Home rats whose final training performances were similar. Pairing was adjusted such that within-pair (i.e. Novel-Home) performance differences in terms of daily rewards did not differ significantly from zero. This matching was critical for adequate assessment of competitive ability because a difference in motivation or in learning to obtain the rewards could confound the measure for competitiveness [83]–[85]. As body sizes could also influence competition results, within-pair weight differences were checked based on measurements made both 2 months prior and 1 month after the competition to ensure that within-pair differences were not significantly different from zero. Although 15 pairs of Novel and Home rats underwent training and competition testing, only 11 pairs met the above constraints, thus behavioral data from only these 11 matching pairs were analyzed. Out of these 11 pairs, 4 pairs were comprised of rats that were littermates.

Pairs were tested on two consecutive days, with 12 trials per day, in the same room where training took place. Competition testing was conducted in the same way as during training with the following exceptions. First, testing occurred in neutral, non-home cages that were clean and lined with fresh bedding to avoid aggressive behavior motivated by territoriality. Second, Novel and Home rats were marked with either red or green food coloring on the sides of their bodies to distinguish the two rats in each pair, with colors counterbalanced between Novel and Home groups. Third, Novel and Home rats were habituated to the testing cage simultaneously for 2 min prior to competition trials. Fourth, a trial was terminated when one of the two rats obtained the reward or when the 30 s upper limit was reached.

### Post-Competition CORT Concentration

The state of HPA activation after social competition testing on each of the two consecutive days and the habituation of HPA response across the two days was assessed by measuring circulating CORT concentration from blood samples obtained via tail nick 15 min after the completion of social competition testing. Blood samples from both rats in each pair were simultaneously collected by two groups of experimenters at two separate stations to ensure similar time delays for both rats. As previously described, Novel and Home identity of rats was marked using red and green food coloring with colors counterbalanced between groups. Thus, the experimenters were blind to rats' group identity. At each station, rats were held gently under a large paper towel by one experimenter and the blood samples were collected by a second experimenter. Samples were centrifuged, and plasma was removed and stored at −20°C until radioimmunoassay was performed. Plasma CORT concentration was measured in duplicate in a single assay using the Coat-a-Count Corticosterone Kit (Diagnostic Products, Los Angeles, CA). The lower limit of detection was 12.4 ng/ml and the intra-assay coefficient of variation was 11.3%.

### Data Analysis

ANOVAs with Novelty and Day as within-factors were performed on training performance, competition wins, and post-competition CORT concentration. For the analysis of training performance, due to the presence of a significant litter effect, litter was used as the unit of analysis. For the analysis of competition wins and post-competition CORT, no litter effects were found, thus pairs of competing rats were used as units of analysis because the two measures from each pair were not independent; furthermore, whether pairs were comprised of littermates or non-littermates was entered into the model as a between-factor. ANOVA with Novelty as a between-factor and Day as a within-factor was performed on activity level; because no litter effect was found, individual rats were used as units of analysis. Following significant interactions or main effects, pairwise and one-sample t-tests were performed [86]. We hypothesized that Novel rats would win more often than Home rats based on a prior finding from younger adult rats [20]; accordingly, directional tests were used for paired t-tests performed on competition data. Wilcoxon signed ranks tests were used to test for differences in retrieval order and average retrieval latency between Novel and Home pups. Pearson correlations between the maternal measures and the novelty scores for each litter (mean for Novel rats–mean for Home rats within a litter) were computed to test the maternal modulation hypothesis. Possible relationships between the male-female composition of litters and measures of maternal care and offspring development were tested by computing Pearson correlations between the number of males kept in each litter and (1) maternal LG average, (2) maternal LG variability, (3) novelty effect on competitive success, and (4) novelty effect on CORT habituation.

For the analysis of neonatal novelty exposure effects on behavioral measures and CORT measures, different numbers of rats were involved. For analysis of behavioral measures, we were constrained by the fact that data from 4 pairs of rats had to be excluded because their final training performance could not be matched. Therefore, only 11 of the 15 pairs were used. For analysis of CORT measures, we included the pairs of rats with non-matching final training performance to maximize the sample size. One pair was excluded because one member of the pair was an outlier in CORT concentration. Therefore, 14 of the 15 pairs were used.

For the analysis maternal modulation of competitive success and CORT habituation, the unit of analysis was litter. Therefore, the Ns for the correlations involving competitive success and CORT habituation were 7 and 11 litters, respectively. This means that the tests for maternal modulation of competitive success are low-powered relative to the tests for maternal modulation of CORT habituation. This power difference may explain why the correlation between maternal care variability and CORT habituation reached statistical significance while the correlation between maternal care variability and competitive success was of a similar direction but did not reach statistical significance.
